# Anatomy

**Published:** 1890-08-09

**Authors:** 


### The Student's Bookshelf.

[Books for Review should be sent to The Editor, The Lodge, Porchester Square, W.]

### ANATOMY.

This work* is the result of a natural and very creditable desire on the part of Mr. Owen to utilise for the benefit of future students the experience gained during a service of twelve years' duration as lecturer on anatomy in one of our large medical schools. In his endeavour to render a lasting and more extended record of this work, the author decided to make his book a thoroughly practical one, and to adapt it to the needs of those who having passed through the regulation courses of methodical teaching, are about to apply the results of such study in the wards and out-patient department of their hospital. The book, indeed, is one of the kind known as treatises on topographical or regional anatomy, but differs somewhat from most of these in referring briefly, though with sufficient fulness and very clearly, to the structure and relations of almost every anatomical constituent of the human body. Mr. Owen lays claim to some credit for courage in having trespassed in his book upon the domains of the physician, but we imagine that in any work on applied anatomy it would not be easy to avoid doing this, and certainly at the present day no hospital surgeon would venture to ignore the relations and situation of any of the internal viscera. The work has, on the whole, been well done, and this manual will doubtless prove of great service not only to senior students, but also to practitioners and others who might desire to renew readily and in a short time their exhausted stock of anatomical knowledge. There are here and there some indications of haste in the composition or correction of some parts of the book, but these, we have very little doubt, will have disappeared in future editions. There is one defect which, though it might be proved by Mr. Owen to be unavoidable, and which certainly is common to most English books of this kind, is really an important one. This occurs in the illustrations, which, both in quality and quantity, compare somewhat unfavourably with those found in many standard works on regional and surgical anatomy by French and German authors.

In one respect Mr. Owen's Manual of Anatomy will be found very interesting. We know of no work better calculated to reveal to the elderly practitioner or general reader the widely extended range that has recently been acquired by modern surgery. In the section on the encephalon the combined results of recent physiological and pathological inquiries, and the influence of these on surgical practice, have been fully and very clearly described. The various lesions of the spine and spinal cord are described at some length, and allusion is made to Horsley's classical case in which a tumour was removed from the cervical portion of the medulla. In the section on the pericardium directions are given for the performance of paracentesis of this sac in cases both of serous effusion and of suppurative pericarditis. Reference is made to gastrostomy, to digital dilatation of the pylorus, to laparotomy for intestinal obstruction and ruptured bladder, and to operations on the gall bladder, the kidney, the cæcum, and the prostate. Although this book, as Mr. Owen points out in his preface, has been written by a practical surgeon, the sections dealing with medical anatomy and pathological lessons of internal organs are complete and very instructive, and the reader is also made acquainted with many points related to the practice of the obstetrician.

* "A Manual of Anatomy for Senior Students," by Edmund Owen, M.B., F.R.C.S., surgeon to St. Mary's Hospital, London, and co-lecturer on surgery (late lecturer on anatomy) in its medical school. With numerous illustrations. (London: Longmans, Green, and Co., and New York, 15, East 16th Street, 1890.)

